# Surgical Tuberculosis*An Inaugural Thesis.

**Published:** 1888-05

**Authors:** T. B. Swartz

**Affiliations:** St. Luke’s Hospital, Chicago


### The Medical Journal and Examiner.

VOLUME LVI. MAY, 1888. NUMBER 5.

### SURGICAL TUBERCULOSIS.*

BY T. B. SWARTZ, A. M., M. D.

Since Koch's announcement of the discovery of the bacillus tuberculosis, in March, 1882, the medical press has devoted more time to the discussion of this subject in all its details than has been given to any other theory in medicine promulgated in recent years; and, certainly, no discovery in the province of medical science in modern times has surpassed it in clinical and pathological value, or provoked such general and acrimonious discussion.

But while the theory of the bacillus tuberculosis has not been accepted by some very able men in the profession, the fact of the existence of this micro-organism and of its importance as an etiological factor in one of the most common and most fatal diseases, has been demonstrated, and is now generally recognized.

It is, however, not my purpose to discuss the efforts made to controvert Koch's theory, but rather to examine briefly the present status of the question, and then to pass on to a brief consideration of the tubercular diseases in which the surgeon is especially interested.

Until comparatively recent years tuberculosis was considered exclusively a local disease of the lungs ; the term *tuberculosis* was as definite in its designation of a disease as the term Pott's disease. But since we now know that nearly every organ or tissue of the body may become the field of action of this virus, the term tuberculosis no longer locates the disease, but indicates only an idea of the histological topography of the lesion, *i. e.*, a nodule with certain definite elements and having a given life-history.

Although the discovery of Koch was at first turned to advantage, chiefly in the diagnosis of lung diseases, thus restricting its value principally to the field of medicine, yet surgery has not failed to benefit by it in a most important degree. The recognition of the surgical value of this discovery has been a matter of slow growth, and the profession to-day, outside of Germany, is by no means a unit on this question.

While Koenig, in Germany, asserts that the surgeon there considers almost every chronic inflammation a tubercular disease, yet one widely read work on diseases of the joints, by an American author, published since 1883, does not mention the subject of tuberculosis, but the author contents himself with the use of the vague terms "strumous and scrofulous" joints. The same may be said of a similar work published in London as late as 1886, to which I may have occasion to refer again.

I shall endeavor to give here only a brief

*An Inaugural Thesis.

*résumé* of the arguments which, in my opinion, render the mycotic origin of the tubercle an established fact in medicine, and in doing this, time and space will permit only a statement of propositions which have been satisfactorily demonstrated:

1. *Is the tubercle infectious?*

Koch has pointed out that Klencke was the first investigator who successfully inoculated animals with human tuberculosis in 1843. Villemin, in 1866, next took up the subject, and not only affirmed the proposition of Klencke, but proved also the converse to be true.

He first attracted general attention to the subject by a series of successful inoculations with tubercular virus, producing in *every case genuine tuberculosis*, and he fortified his conclusions by accompanying these inoculations with another series of carefully conducted control-experiments with non-tubercular material *without producing tuberculosis in a single instance.*

Koch, Cohnheim, and others, supplemented these investigations by numerous experiments, with perfect safeguards against error, and *invariably* produced the disease with *tubercular virus* and *never with any other*. Immediately upon the publication of the results of these experiments, opposition began to develop. Among the most prominent critics may be named Burdon Sanderson, Wilson Fox, and others of note.

They attacked the question negatively, and by numerous experiments sought to vitiate the conclusions of these investigators by showing that tuberculosis could be produced by other than tubercular material.

They succeeded for a time in raising a doubt as to the accuracy of the foregoing conclusions, but when their methods of experimenting became known it was evident that they had failed to surround their work with the necessary safeguards against error; that their work was practically unreliable and without value, and Koch's conclusions stand to-day in no immediate danger of cantradiction.

We may therefore accept it as established:

I. That tuberculosis is infectious and can be propagated through a *series* of animals by inoculations, and that no matter how remote the inoculation, the disease retains its identity and infectious character; and,

II. That the disease *cannot* be produced by inoculation, inhalation, or by any other method with any other material, organic or inorganic.

2. *What is the infectious element?*

When Koch had been convinced of the truth of these propositions he set to work to discover, if possible, the particular element in the tubercle which is the cause of infection. The native histological elements of the tubercle were then, as now, well understood, and since all of them occur in ordinary granulation-tissue, identical in every particular except that of arrangement, and yet when taken from such tissue are perfectly innocuous, he was forced to the conclusion that the infectious property of the tubercle lay in some element not yet discovered—perhaps some specific poison of a parasitic nature foreign to the body.

A full description of Koch's work in this line of thought is not essential here, and I will state only the points which he sought to establish.

They are as follows:

I. To determine whether in tubercular tissues elements occur foreign to the tissues and not produced by them.

II. Having found such elements, to prove, if possible, that they are living organisms having differentiating characteristics and capable of indefinite multiplication.

III. To show that tissues containing such organisms, when used for inoculation would produce the disease and that no other tissues would do so.

IV. Whether, by cultivating this organism in suitable soil until a *perfectly pure* culture had been obtained, he could pro_

duce the disease with such culture by inoculation.

V. To show by control-experiments that inoculation by any other substance not containing such an organism *never does* produce the disease.

All this he successfully accomplished with marvelous accuracy, and by experiments extending over hundreds of cases and with uniform results.

This organism, having distinct characteristics and capable of ready differentiation from all other forms of schizomycetes, Koch calls the "Bacillus tuberculosis," and in it resides the efficient etiological factor of tuberculosis. Without entering into further detail we will assume as demonstrated the two propositions pertaining to the etiology of the tubercle and its infectious character. If we now keep in view the well-known histological elements of the tubercle, together with the propositions just stated, we will be able to define a tubercle as a cellular, non-vascular nodule containing the bacillus tuberculosis, and varying but little in its histological elements and life-history.

As a basis of some remarks on the surgical treatment of tuberculosis, we may consider briefly a few facts in the pathology of the tubercle. Since the bacillus tuberculosis is the irritant or cause of this disease, and is constantly escaping from tubercular tissues and floating freely in the air, why is it that we do not all become infected? Why do the few alone suffer? Obviously the most rational explanation lies in the varying susceptibility of different individuals, and of different tissues in the same individual. Therefore, in addition to the presence of the specific irritant there must be also a specific susceptibility—that is, a suitable soil and favorable conditions of temperature, rest, and moisture.

The bacillus tuberculosis gains access to the body by means of the air or food, and having lodged, the soil being suitable, the germ proceeds to multiply in loco. The presence of the germ in the tissues or the products which it evolves is a mild chronic irritant and causes corresponding inflammation. The products of inflammation gather around the focus of irritation and take the special structural arrangement which is readily recognized under the microscope, and which is so well understood that it needs no description here. This structure has, however, a special tendency to necrosis, the one usually affecting albumenoid structures, namely fatty degeneration terminating in caseation. This degeneration is as characteristic of the tubercle as any element which it contains; it is an invariable factor in its history.

The bacilli are carried, probably by the leucocytes, into contiguous parts, and a series of secondary foci spring up around the primary focus, passing through similar stages toward necrosis, and from proximity blend with the primary focus; thus the disease spreads by means of progressive local infection. There may thus result the destruction of a part or the whole of an organ, and the disease still be a local one. But at any moment the bacilli may find their way into the lymph-channels and through them into the blood, or they may enter more directly by penetrating the vessel walls; and when this occurs the disease is no longer local, but every part of the body becomes liable to its destructive influences and we have a general disease, acute miliary tuberculosis.

This brief pathological history should be of interest to the surgeon for the following reasons:

1. The disease is infectious.

2. It spreads from a single focus to adjoining parts, and entering the blood becomes a general disease terminating in death. It is malignant in the sense that it is metastatic. It may not be as fatal as cancer when not interfered with, but the record of cases where the primary focus when fully developed has become encapsuled, calcified, or otherwise mysteriously disappeared, is so small that this element may be practically eliminated from consideration in making our prognosis of the case, or in determining on a mode of procedure in its treatment. The case may be stated dogmatically as follows: Given a primary tubercular focus within the field of surgical operations, it is the surgeon's imperative duty to eradicate it before general infection occurs and a fatal result is inevitable. Perhaps all surgeons of the present day are willing to subscribe to this principle, but undoubtedly there exists a great diversity of opinion as to the etiology and pathology of many surgical diseases that are unmistakably tubercular.

While tubercular lesions of various tissues of the body besides the lung were early recognized by Delpech, Nichet, Nelaton, and others, their knowledge was based wholly upon the histological elements of the tubercle. Since the discovery of Koch, however, Volkmann, Friedländer, Koenig, and others, have used the microscope so energetically and so efficiently on a great variety of lesions, and have found the bacillus tuberculosis invariably in certain classes of diseases, that they are now quite generally considered as being always of tubercular origin.

I have already adverted to the prevalence of this opinion in Germany, but in a work entitled "Diseases of the Joints," by Howard Marsh, of London, published in 1886, no reference is made to tubercular disease of the joints, although the author devotes considerable space to the consideration of "Scrofulous Diseases of the Joints," and of "Strumous Joints." He gives, however, as "complications" of these affections, unless early and successfully treated, phthisis, tubercular meningitis, etc. Thus it would seem that while the profession is practically a unit as to the treatment of tubercular lesions, when recognized, there appears to be want of recognized pathognomonic signs of such lesions when they occur.

As long as the terms "strumous joint" and "scrofulous disease" are retained, there will probably continue to be confusion and error in diagnosis, for they represent only a condition, not a disease.

If what has been said be true, then it is of vital importance in these cases that early and correct diagnosis be made; and in proportion as the surgeon depends upon the microscope in diagnosis will scrofula disappear from the list of pathological lesions, and application of the term become restricted to designation of a condition of the system—a constitutional diathesis which predisposes to tubercular disease. The correctness of this definition of scrofula has been demonstrated by conclusive investigations. The term should be used only to designate a depraved constitution of low vitality, one especially susceptible to turbercular infection.

M. Rendu states the case as follows: "Scrofula and turberculosis bear the same relation to each other as soil to seed." If chronic inflammation occur in such an individual it seems safe to conclude that it is tuberculosis.

Regarding tuberculoses occurring within the province of surgery, time will not permit anything like a complete review of the history, pathology, diagnosis, and treatment of these diseases, and necessarily consideration of that phase of the subject must be general rather than special, and under the following divisions:

I. Diagnosis.

II. Treatment.

III. Enumeration of surgical tuberculoses.

I. Diagnosis.

*a.* Presumptive.

*b.* Positive.

By presumptive diagnosis is meant diagnosis based upon the history of the case, together with its physical characteristics, and the general condition of the patient. By positive diagnosis is meant that based upon the microscope, upon inoculations, or both. What degree of probability or positiveness may attach to presumptive diagnosis, as defined above?

Although positive diagnosis of a tubercular lesion must necessarily be based upon microscopic examination or inoculation-experiments, with due safeguards against error, yet the constancy with which the disease appears in certain organs or tissues, under certain well-defined conditions, and manifesting itself with more or less constant characteristics, gives to such a presumptive diagnosis a degree of probability which makes it differ from a positive diagnosis by less than any assignable quantity. Thus tubercular hip-disease, Pott's disease, necrosis of bone with rarefaction, occurring in the so-called scrofulous individuals, are always regarded as being tubercular in orign. But the same can not be said of all tubercular lesions, and the microscope is frequently the only reliable means of diagnosis. The importance of early diagnosis in these cases leaves no room for doubt as to the duty of the surgeon in regard to the use of the microscope in all forms of chronic suppurative inflammation.

Negative results in its use in unskilled hands do not imply that positive results are not possible in the hands of those more expert in the use of the instrument. The microscope of a magnifying power of, at least, from 300 to 500 diameters is recommended; Koch advises 500 to 700 diameters, with an Abby condenser for thick sections. The sections must have a special staining, and the process known as Ehrlich's modification of Koch's method is, perhaps, the most accurate and convenient. This method of making a diagnosis may seem long and troublesome, but it does not consume much time where all conveniences are at hand.

Nowhere is the bacillus tuberculosis found in such large numbers as in the lungs. The sputum of a tubercular patient usually contains the bacilli in sufficient numbers to enable the microscopist to detect them with comparative ease. It is not so, however, in tubercular lesions of other parts of the body. Frequently but a single bacillus can be found in such a section, and it is often necessary to examine a large number of slides before the germ can be detected. Koch, at one time, examined forty sections from a lupus before he found the germ.

II. Treatment.

Of the treatment, in general, but little need be said. From the nature of the disease it must be radical and early in order to be efficient. The indications are—

1. Medical, to attempt to build up the system and fortify the tissues against further invasion.

2. Surgical, to eradicate the focus as early as possible.

These indications become self-evident from the infectious and metastatic character of the disease, *i. e.*, it is fatal without early and adequate treatment.

It is not to be inferred that every case of tuberculosis can be cured by operative measures. Sometimes the disease, like carcinoma, recurs. This is readily explained upon one of two hypotheses—either the focus removed was not the primary focus, but a secondary or metastatic lesion, and the primary focus still remains as a source from which secondary lesions continue to develop; or, if the primary focus was operated upon, it was not completely eradicated and it continues to spread. Indeed, Koenig has estimated that of all tubercular diseases of bones and joints, 79 per centum are secondary lesions. Nevertheless, when the disease manifests itself in such a way that its diagnosis becomes positive, surgical interference is the only rational treatment, and it alone can hold out hope of permanent recovery of the patient.

III. Enumeration of surgical tuberculoses.

Only some of the more important of these lesions will be considered here.

*a. Tuberculosis of bones.*

That this tissue is especially liable to tubercular infection, has been demonstrated. So constantly is the bacillus tuberculosis found in *ostitis rarefaciens* that many surgeons consider caries of bone as being always tubercular in character. This is perhaps a radical position to assume, but I believe that it results in less harm to patients than the extremely conservative position of denying the frequent occurrence of tuberculosis, and putting patients on an expectant plan of treatment.

Caries of bone is well defined by Boyd as an erosion of bone by tubercular inflammation, syphilis being almost the only other cause. Traumatism may be a secondary cause; its mode of operation will be referred to later. Caries attacks spongy bones far more often than compact ones, the vertebræ being by far the most commonly affected, as in the condition known as Pott's disease.

Chronic suppuration is nearly always connected with this form of bone-disease, and is, to some extent, characteristic. Often it is profuse, seeking the surface in the line of least resistance. The chief danger lies in exhaustion, septicæmia, or acute tuberculosis. These lesions occur most frequently in children, and notably in those possessing the so-called scrofulous constitution.

The treatment has already been indicated. The general condition must first receive attention; then, if possible, the tubercular focus should be thoroughly eradicated by surgical means. In the case of Pott's disease, radical operations, of course, are impossible, but much can be done by change of climate and constitutional treatment. Dr. Edmund Andrews has had excellent results by dissolving out the carious bone with dilute hydrochloric acid of such strength as the patient can bear. His mode of treatment, together with the results of numerous and careful experiments with various bone-solvents, are fully explained in a paper read before the American Medical Association, June, 1887.

b. *Tuberculosis of Joints.*

This is a frequent disease, especially among children.

Recently pathologists have come to view joint-disease as primarily tubercular, and that traumatism, while it may be an exciting or secondary cause, is seldom, if ever, the primary etiological factor. The manner in which the joint becomes infected is of interest.

Tuberculosis of a joint is usually a secondary lesion, the primary focus being, perhaps, a neighboring caseous gland, or the source of infection may be located in the cancellous tissue of the proximal end of one of the bones forming the joint. A knee-joint may be primarily attacked by tuberculosis without any other focus; but it has been shown that a primary source of tubercular arthritis of the knee has been a focus situated in the cancellous tissue of the lower epiphysis of the femur, the caseous products of this focus having found their way into the joint with resulting infection. When a joint, situated near such primary foci, suffers from traumatism, there is set up a current of lymph and blood in the direction of the injured joint; there is determination of fluids to this part; hyperæmia, and the caseous *débris* of such a tubercular lymph-gland, together with the contained bacilli, is swept along with the current into the joint and the result is tuberculosis following traumatism. If the injury be a compound fracture, infection may come from the atmosphere. The hip-joint is especially liable to disease of tubercular character and "disease of the hip-joint" usually means tuberculosis, although this joint like all others, is liable to inflammation having other origin. Outside of tuberculosis of the lungs, tuberculosis of the hip-joint is most unfavorable as regards prognosis; this is especially true if the disease go on to suppuration.

Of 401 cases of hip-joint disease reported from the Hospital for Hip-joint Diseases, London, in 69 per centum there was suppuration.

The Alexandrian Hospital, London, reports 260 cases of suppurative hip-joint diseases, of which 31.6 per centum proved fatal, the cause of death being chiefly tuberculosis acute or local.

The rational treatment seems to be excision, and yet the mortality resulting from the operation has been very large, but improved methods of operating, greater skill, and better antisepsis, in the future may materially reduce this high death-rate.

It may be stated in conclusion that all bones and all joints of the body are liable to infection, for, under diseased conditions, the bacillus tuberculosis has been found in most of them.

*c. Other tuberculoses.*

Some of the tissues and organs of the body which have been the seat of tuberculosis, and in which the bacillus tuberculosis has been demonstrated, are given as follows:

Abcess, pre-sternal and costal, by Debove; Abcess, cold, by Schuckhart and Krause; Bladder, by Wesener; Epiglottis, by Voltolini; Epididymis, by Brouilly; Fallopian tubes, by Koch; Fungoid ostitis of rib, by Brouilly; Genito-urinary tract, male and female, by Koster; Glans penis, by Koch; Intestines, by Koch; Kidney, by Gondolphe; Lymphadenomata, by Schüppel; Lymphatics, cervical, by Guarneri; Lupus, by Friedländer and Koch; Larynx, by Koster; Mucous membrane ulcers, by Volkmann; Mouth, by Volkmann; Mammary cysts, by Friedländer; Os uteri, by Friedländer; Osteo-myelitis, by Kanzler; Ovary, by Koch; Ozena scrof., by Volkmann; Pharynx, by Voltolini; Rectal fistula, by Voltolini; Skin, by Friedländer; Supra-renal capsules, by Koch; Tongue, by Koster; Tonsils, by Guttmann; Tendon-sheaths, by Koenig; Testicles, by Schuckhart and Krause; Uterus, by Koze and Simon; Urethra, by Koch; Velum palati, by Guttmann; Vagina and vulva, by Wesener.

The following books and periodicals have been consulted in the preparation of this paper, and they present a partial bibliography of the subject:

BIBLIOGRAPHY.

Micro-Organisms and Disease, E. Klein, M. D., F. R. S., London; Pathological Anatomy, Billroth (12th German edition); Pathology, Ziegler; Manual of Pathology, Joseph Coats, M. D; Pathology and Morbid Anatomy, Green; Diseases of Bones and Joints, Koenig, Vol. III (German edition); Diseases of the Joints, Howard Marsh, M. D., F. R. C. S., London, 1886; Druitt's Vade-Mecum, Stanley Boyd, M. D., F. R. C. S., London, 1887; Text Book of Surgery, John A. Wyeth, M. D., New York, 1887; Practice of Surgery, Thomas Bryant, M. D., F. R. C. S., 1885; Surgical Tuberculosis, R. H. Hall, M. D., N. Y. Med. Journal, 1885, Jan. 17; Removal of Necrosed Bone by Irrigation with weak Hydrochloric acid, Edmund Andrews, M. D., LL. D., Jour. Am. Med. Ass., Aug. 13, '87; Relation between Tubercular Joint Disease and General Tuberculosis, F. S. Dennis, M. D., N. Y. Med. Journal, Dec. 27th, 1884; Buccal Tuberculosis, Byron Delavan, M. D., N. Y. Med. Jour., May 14th, 1887.

ST. LUKE'S HOSPITAL, CHICAGO.

